# Adult patients with ACL tears have greater tibial internal rotation in MRI compared to adolescent patients

**DOI:** 10.1186/s13018-022-02912-0

**Published:** 2022-01-11

**Authors:** Chih-Kai Hong, Yu-Ju Lin, Ting-An Cheng, Chih-Hsun Chang, Kai-Lan Hsu, Fa-Chuan Kuan, Wei-Ren Su

**Affiliations:** 1 Department of Orthopaedic Surgery, National Cheng Kung University Hospital, College of Medicine, National Cheng Kung University, No. 138, Sheng-Li Road, Tainan City, 70428 Taiwan; 2 Skeleton Materials and Bio-Compatibility Core Lab, Research Center of Clinical Medicine, National Cheng Kung University Hospital, College of Medicine, National Cheng Kung University, Tainan City, Taiwan; 3 Department of Biomedical Engineering, National Cheng Kung University, Tainan City, Taiwan; 4 Musculoskeletal Research Center, Innovation Headquarter, National Cheng Kung University, Tainan City, Taiwan

**Keywords:** Anterior cruciate ligament, Anterior tibial translation, Tibial internal rotation

## Abstract

**Purpose:**

To compare the anterior translation and internal rotation of tibia on magnetic resonance imaging (MRI) between adult and adolescent patients with anterior cruciate ligament (ACL) tears.

**Methods:**

Patients who underwent isolated ACL reconstruction from January 2013 to May 2021 were retrospectively reviewed. The exclusion criteria included incomplete data, poor image quality, a prior ACL surgery, and concomitant fractures or other ligament injuries. The enrolled patients were divided into two groups based on their ages: an adult group (age > 19 years) and an adolescent group (15 to 19 years of age). Anterior tibial translation and femorotibial rotation were measured on MRI. A Student’s t-test was used for the statistical analysis comparing the adult and adolescent groups.

**Results:**

A total of 365 patients (279 adults and 86 adolescents) were enrolled in the present study. The anterior tibial translation in the adult group (4.8 ± 4.4 mm) and the adolescent group (5.0 ± 4.2 mm) was not significantly different (*p* = 0.740). On the other hand, the tibial internal rotation in the adult group (5.6 ± 5.0 degree) was significantly greater compared to the adolescent group (4.2 ± 5.6 degree) (*p* = 0.030). The intraclass correlation coefficients (ICC) of the measured data from two independent observers showed excellent reliability (0.964 and 0.961 for anterior tibial translation and tibial internal rotation, respectively).

**Conclusion:**

The adult patients with ACL tears exhibited significant greater tibial internal rotation compared to the adolescent patients, whereas the magnitude of the anterior tibial translation was similar in both groups. Care should be taken if clinicians plan to establish the cutoff point values for diagnosis of ACL tears using the femorotibial internal rotation angle.

## Introduction

The anterior cruciate ligament (ACL) is a structure that contributes to the restraints of anterior translation and internal rotation of the tibia relative to the femur [1–6]. In addition to the integrity of the ACL itself, several additional signs have been proposed that can assist with the diagnosis of ACL tears on magnetic resonance imaging (MRI), including anterior tibial translation [7, 8], internal rotation of the tibia [6], the lateral femoral notch sign [9, 10], and bone kissing contusions [10, 11].

Several studies have indicated that ACL-deficient knees feature static instability parameters on MRI [4, 6, 8, 12]. Anterior translation of the tibia has been reported to be greater in ACL-deficient knees compared to healthy knees [8, 12]. Numkarunarunrote et al. further proposed cutoff distances of 3.5 mm and 5.5 mm for diagnosis of partial and complete ACL tears [8]. Meanwhile, the magnitude of tibial rotation on MRI in patients with ACL tears has also been evaluated. Vassalou et al. demonstrated an increase in tibial internal rotation in adult patients with ACL tear [6], whereas Mitchell et al. found a greater internal tibial rotation in adolescent ACL-deficit knees [4].

To our knowledge, there is a paucity of data that compares image features of ACL-deficient knees on MRI images between adults and adolescents. The suggested cutoff values obtained from adult patients for diagnosis of ACL tear may not be applicable in adolescent patients. Therefore, the purpose of the present study was to compare the anterior translation and internal rotation of tibia on MRI images in adult and adolescent patients with ACL tears. We hypothesized that the magnitude of tibial internal rotation in ACL-deficient knees is different between adults and adolescents.

## Methods

After Institutional Review Board approval (ID No. A-ER-110-437) in a tertiary medical center, charts were retrospectively reviewed for all patients undergoing isolated ACL reconstruction at a medical center from January 2013 to May 2021. Patients were excluded if the data were incomplete, if image quality was poor (slice thickness > 3 mm), if there was a prior ACL surgery, or if there were any concomitant fractures or other knee ligament injuries. Demographic data consisted of age, gender, and laterality. The patients were divided into two groups based on their ages: an adult group (age > 19 years) and an adolescent group (15 to 19 years of age).

MRI images of each included patients were reviewed using a radiology information system (INFINITT PACS, INFINITT Healthcare Co. Ltd., South Korea). The MRI was performed on a 1.5-T MRI scanner (GE Discovery MR450 1.5T Scanner) with the knees in 10 to 15 degree of flexion and neutral rotation. About 10% of the included patients had their MRI images taken at outside institutions, and these images were used in this study only if in-house imaging was not available.

**Fig. 1:**
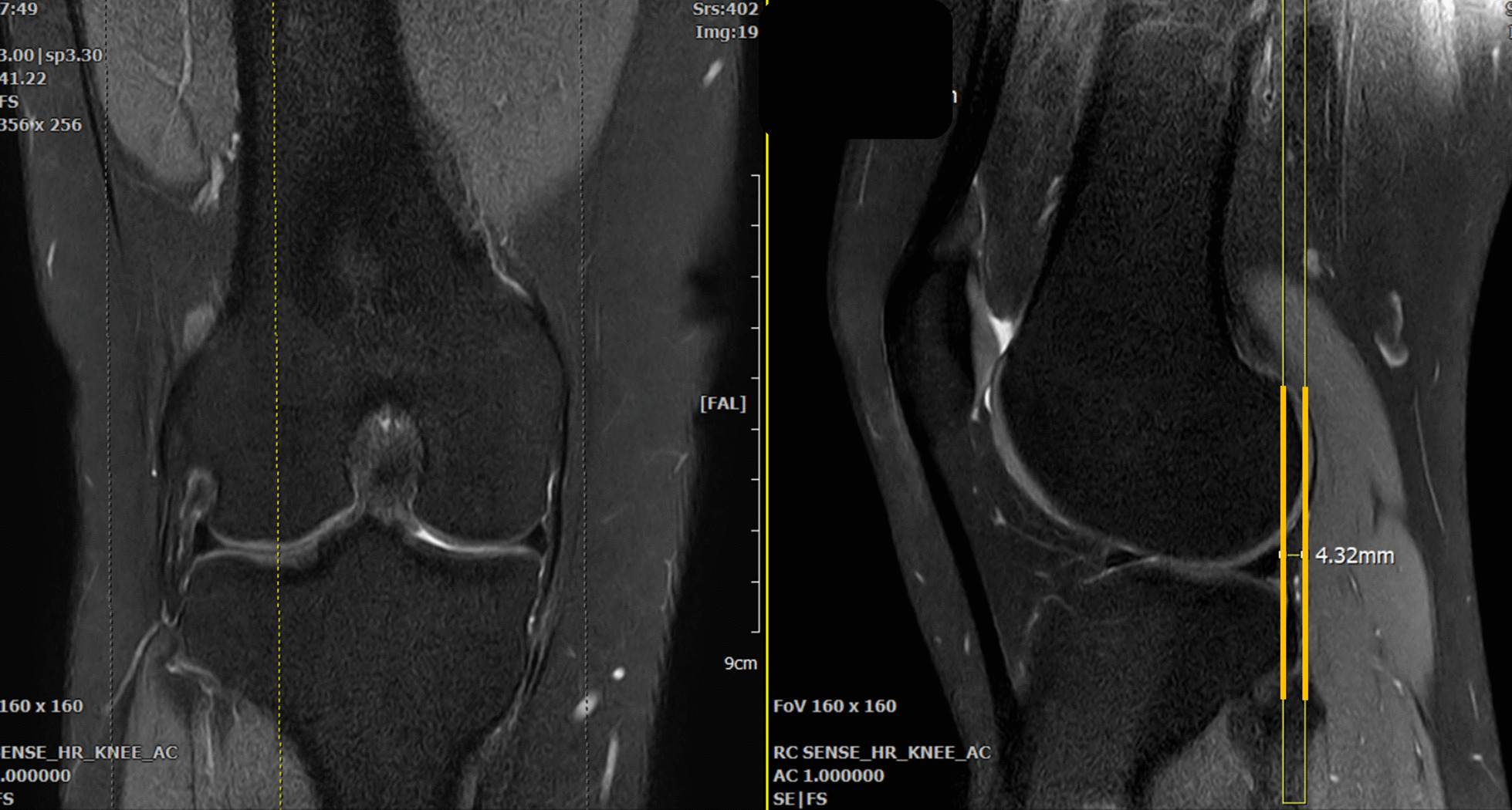
The anterior tibial translation was measured in accordance with the methods described by Kalegowda et al. [13]. The midsagittal plane of the lateral femoral condyle was determined with the assistance of coronal images. The distance between the vertical lines drawn from the posterior aspects of the subchondral bone of the lateral femoral condyle and lateral tibial condyle was measured

The tibial translation on MRI was measured in accordance with the methods described by Kalegowda et al. [13]. The proton density-weighted images in the sagittal plane were used, and the midsagittal plane of the lateral femoral condyle was determined with the assistance of coronal images. Two vertical lines were drawn from the posterior aspects of subchondral bone of the lateral femoral condyle and lateral tibial condyle, respectively. The shortest distance between the two vertical lines was considered to be the tibial translation, which was given a positive value in case of anterior translation and was given a negative value in cases of posterior translation (Fig. 1).

**Fig. 2:**
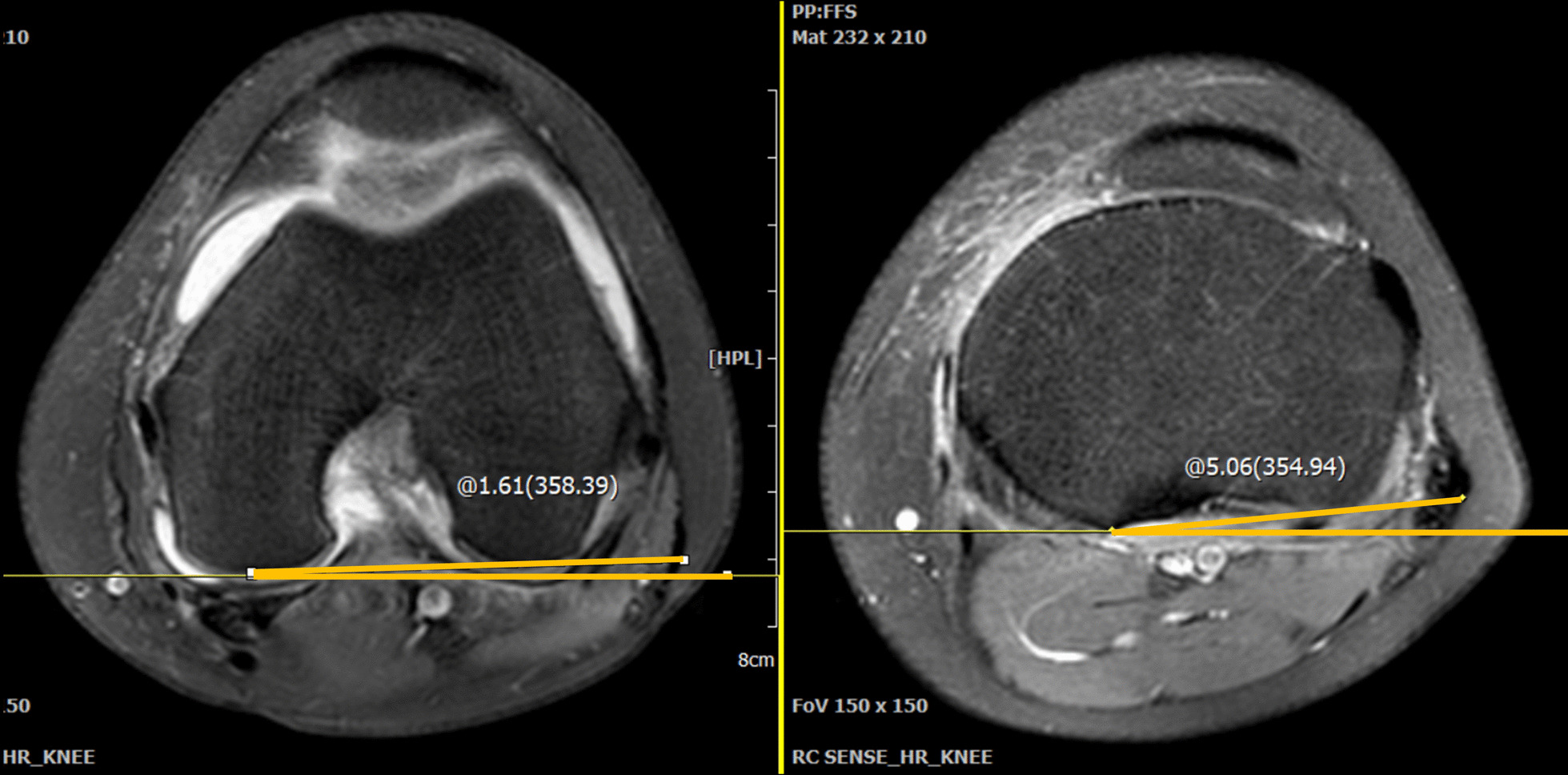
The femorotibial rotation was measured from images in the axial plane. The angle of the posterior femoral condyles was measured from their most prominent point relative to the horizontal line. Meanwhile, the image that was a cut above the fibular head was chosen, and the angle of the posterior tibia condyles was measured off a horizontal line. The femorotibial rotation was calculated by subtracting the femoral rotation from the tibial rotation

Femorotibial rotation on MRI images was measured using the method described by Vassalou et al. [6]. The proton density-weighted images in the axial plane were used. Femoral rotation was measured from the image that revealed the most prominent point on the posterior femoral condyles, whereas the tibial rotation was measured from the axial image that was just above the proximal end of the fibula. The femoral rotation angle was measured from the tangent line of the posterior femoral condyles and the horizontal line, whereas the tibial rotation angle was measured from the tangent line of the posterior cortex of the tibial condyles and the horizontal line. The femorotibial rotation angle was calculated as the difference between the femoral rotation and the tibial rotation angles. A positive value was given in cases with tibial internal rotation, whereas a negative value was given in cases with tibial external rotation (Fig. 2).

The statistical analysis was performed using IBM SPSS Statistics (version 22; IBM SPSS Inc., Chicago, IL, USA). The Shapiro–Wilk test was used to evaluate whether a variable was normally distributed, whereas the Levene test was used to evaluate homogeneity of variance. Descriptive statistics, including means and standard deviations, were obtained for both groups. A Student’s t-test was used to identify statistically significant between-group differences. The intraclass correlation coefficients (ICC) were calculated to determine the interobserver reliability for the measured variables. A *p* ≤ 0.05 was considered statistically significant.

## Results

A total of 489 patients met the primary inclusion criteria. After evaluation, 32 patients were excluded due to revision ACL reconstruction, 26 patients were excluded due to multi-ligament injuries, and 66 patients were excluded due to poor image quality, MRI slice thickness greater than 3 mm in the sagittal plane. Therefore, a total of 365 patients were finally included in the present study. Two hundred and seventy-nine patients (213 males and 66 females) with a mean age of 30.7 ± 9.4 years were included in the adult group. Meanwhile, eighty-six patients (50 males and 36 females) with a mean age of 17.3 ± 1.4 years were included in the adolescent group. The laterality between the adult group (133 right and 166 left knees) and the adolescent group (32 right and 54 left knees) was not significantly different (*p* = 0.107).

The results of the Shapiro–Wilk tests showed that the data on the anterior tibial translation and tibial internal rotation were normally distributed. Therefore, the parametric method, a Student’s t-test, was chosen for the statistical analysis. The anterior tibial translation in the adult group (4.8 ± 4.4 mm) and the adolescent group (5.0 ± 4.2 mm) were not significantly different (*p* = 0.740). On the other hand, the tibial internal rotation in the adult group (5.6 ± 5.0 degree) was significantly greater as compared to the adolescent group (4.2 ± 5.6 degree) (*p* = 0.030). The ICC of the measured data from two independent observers showed excellent reliability (0.964 and 0.961 for anterior tibial translation and tibial internal rotation, respectively).

## Discussion

Both anterior translation and internal rotation of the tibia have not only been proven to be related to ACL-deficient knees and but have also been considered to be secondary signs of ACL tears [6–8]. To our knowledge, it remains unclear whether the aforementioned parameters are different between adult and adolescent ACL-deficient knees. Therefore, the current study was aimed toward comparing the anterior translation and internal rotation of the tibia on MRI images in adult and adolescent patients with ACL tears. The major finding of the present study was that the adult patients with ACL tears had significantly greater tibial internal rotation compared to the adolescent patients.

Anterior tibial translation has been recognized as an indirect sign of an ACL tear [4, 8, 12]. Numkarunarunrote et al. indicated that anterior tibial translation was significantly greater in patients with complete ACL tear compared to patients with intact ACL [8]; they also provided cutoff point distances of 3.5 mm and 5.5 mm for diagnosis of partial and complete ACL tears, respectively. Mitchell et al. evaluated the anterior tibial translation in adolescents and reported that the ACL-deficient cohort had significantly greater anterior translation than the ACL-intact group [4]. In the present study, the anterior tibial translation on MRI in both adult and adolescent patients with ACL tears was further investigated. It was found that the magnitude of the anterior tibial translation was similar in the adults and adolescent ACL-deficient knees. It was considered that the cutoff point distances for diagnosis of ACL tear established from adult patients [8] may possibly be appropriate for adolescent patients.

In some previous studies, the magnitude of tibial rotation on imaging in patients with ACL tears was evaluated [4, 6]. Vassalou et al. indicated that adult knees demonstrated a mean of a 7° increase in internal rotation when the ACL was ruptured [6]. They also suggested that a femorotibial angle ranging from 4.9° to 5.5° indicates a complete acute ACL tear [6]. Mitchell et al. investigated the tibial rotation angles in adolescent patients for ACL-intact and ACL-deficient knees [4]. Similar to adults, Mitchell et al. found a significant increase in tibial internal rotation in ACL-deficient knees compared to intact knees [4]. In the present study the tibial internal translation on MRI was further compared in adult and adolescent patients with ACL tears. Surprisingly, it was found that adult patients with ACL tears exhibited significantly greater tibial internal rotation compared to adolescent patients. Since the prevalence of concomitant knee injuries was greater among older patients with ACL tear [14], we suspected that the preserved knee structures in adolescents with ACL tears possibly prevents tibial internal rotation. Furthermore, caution should be taken if clinicians plan to establish the cutoff point values for diagnosis of ACL tears using the femorotibial internal rotation angle since adult and adolescent patients have different magnitudes of tibial internal rotation.

The present study has several strengths. First, in the current study, image features on MRI images of adult and adolescent patients with ACL tears were compared, the results of which suggested that the cutoff values should be different in these two distinct groups when using tibial internal rotation as a supplement tool for diagnosis of ACL tear. Second, the data from the current study were collected from a single medical center, and the excluded data only represented a small proportion of the potential study sample. Effects of gender, laterality, and race were not found. Third, two orthopedic surgeons measured all of the parameters on MRI, and the ICC of their measurements showed excellent agreement. Therefore, the measured data and analyzed results in the present study were considered reliable.

The current study also had some limitations. First, only patients with ACL complete tears were enrolled in the present study. Since relevant results from healthy controls were not included, the cause of the smaller tibial internal rotation in adolescent ACL-deficient knees could not be verified. Second, the sample sizes of adult and adolescent groups were uneven. Despite this, the sample size in both groups was greater than 30 and thus was considered sufficient for the central limit theorem [15]. Third, the present study evaluated the tibial translation and rotation in ACL-deficiency knees only; therefore, the effect of other structural injuries, such as meniscus tears and anterolateral ligament (ALL) injuries, could not be evaluated. Fourth, it remained unclear whether copers and noncopers had different image characteristics on MRI. Since copers and noncopers use different landing techniques to limit anterior tibial translation [16], further studies will be needed to investigate and compare the image characteristics of these two groups.

## Conclusion

The adult patients with ACL tears exhibited significant greater tibial internal rotation compared to the adolescent patients, whereas the magnitude of anterior tibial translation was similar in both groups. Care should be taken if clinicians plan to establish the cutoff point values for diagnosis of ACL tears using the femorotibial internal rotation angle. Studies that investigate the biomechanical and anatomical differences between adult and adolescent healthy knees will possibly be needed in the future.

## Data Availability

The datasets used and/or analyzed during the current study are available from the corresponding author on reasonable request.

## Abbreviations

ACL: Anterior cruciate ligament
MRI: Magnetic resonance imaging
